# Maternal Exposure to Disinfection By-Products and Risk of Hypospadias in the National Birth Defects Prevention Study (2000–2005)

**DOI:** 10.3390/ijerph17249564

**Published:** 2020-12-21

**Authors:** Ibrahim Zaganjor, Thomas J. Luben, Tania A. Desrosiers, Alexander P. Keil, Lawrence S. Engel, Adrian M. Michalski, Suzan L. Carmichael, Wendy N. Nembhard, Gary M. Shaw, Jennita Reefhuis, Mahsa M. Yazdy, Peter H. Langlois, Marcia L. Feldkamp, Paul A. Romitti, Andrew F. Olshan, The National Birth Defects Prevention Study

**Affiliations:** 1 Department of Epidemiology, Gillings School of Global Public Health, University of North Carolina at Chapel Hill, Chapel Hill, NC 27599, USA; luben@email.unc.edu (T.J.L.); tania.desrosiers@unc.edu (T.A.D.); akeil@unc.edu (A.P.K.); larry.engel@unc.edu (L.S.E.); andy_olshan@unc.edu (A.F.O.); 2 New York State Department of Health, Bureau of Environmental and Occupational Epidemiology, Albany, NY 12237, USA; adrian.michalski@health.ny.gov; 3 Department of Pediatrics, Stanford University School of Medicine, Stanford, CA 94305, USA; suzanc@stanford.edu (S.L.C.); gmshaw@stanford.edu (G.M.S.); 4 Arkansas Center for Birth Defects Research and Prevention, Department of Epidemiology, Fay W. Boozman College of Public Health, University of Arkansas for Medical Sciences, Little Rock, AR 72205, USA; wnnembhard@uams.edu; 5 National Center on Birth Defects and Developmental Disabilities, Centers for Disease Control and Prevention, Atlanta, GA 30333, USA; nzr5@cdc.gov; 6 Massachusetts Department of Public Health, Boston, MA 02108, USA; mahsa.yazdy@mass.gov; 7 Division of Epidemiology, Human Genetics, and Environmental Sciences, University of Texas School of Public Health, Austin, TX 78701, USA; peter.langlois@uth.tmc.edu; 8 Department of Pediatrics, University of Utah School of Medicine, Salt Lake City, UT 84108, USA; marcia.feldkamp@hsc.utah.edu; 9 Department of Epidemiology, College of Public Health, The University of Iowa, Iowa City, IA 52242, USA; paul-romitti@uiowa.edu

**Keywords:** disinfection by-products, hypospadias, birth defects, epidemiology

## Abstract

The purpose of this study was to estimate the association between 2nd and 3rd degree hypospadias and maternal exposure to disinfection by-products (DBPs) using data from a large case-control study in the United States. Concentration estimates for total trihalomethanes (TTHMs), the sum of the five most prevalent haloacetic acids (HAA5), and individual species of each were integrated with data on maternal behaviors related to water-use from the National Birth Defects Prevention Study (NBDPS) to create three different exposure metrics: (1) household DBP concentrations; (2) estimates of DBP ingestion; (3) predicted uptake (i.e., internal dose) of trihalomethanes (THMs) via ingestion, showering, and bathing. The distribution of DBP exposure was categorized as follows: (Q1/referent) < 50%; (Q2) ≥ 50% to < 75%; and (Q3) ≥ 75%. Logistic regression was used to estimate adjusted odds ratios (aORs) and 95% confidence intervals (CIs). Generally, null associations were observed with increasing TTHM or HAA5 exposure. An increased risk was observed among women with household bromodichloromethane levels in the second quantile (aOR: 1.8; 95% CI: 1.2, 2.7); however, this association did not persist after the inclusion of individual-level water-use data. Findings from the present study do not support the hypothesis that maternal DBP exposures are related to the occurrence of hypospadias.

## 1. Introduction

Hypospadias is a structural birth defect of the male urethra that occurs when the urethra is located below its normal location (i.e., tip of the penis). It is a common birth defect in the United States with an estimated prevalence of 64.7 cases per 10,000 male live births [1]. Although this birth defect has a genetic component [2], the majority of hypospadias cases are still considered to be idiopathic [3]. Although the etiology of hypospadias is not well understood, it is hypothesized that the condition may be partially caused by lifestyle, environmental exposures, and gene–environment interactions [4,5,6].

Chlorine is a common disinfecting agent added to public water systems (PWSs) to control waterborne infections. However, chlorine reacts easily with organic substances in surface waters to create other contaminants known as disinfection by-products (DBPs). Two of the most common DBPs in chlorinated water sources, trihalomethanes (THMs) and haloacetic acids (HAAs), are currently regulated by the United States Environmental Protection Agency (USEPA). Currently, the maximum allowable levels for total trihalomethanes (TTHMs) and the five most prevalent haloacetic acids (HAA5s) are 80 and 60 μg/L, respectively [7]. Public health concerns have been raised regarding the association between these contaminants and adverse reproductive outcomes, given that toxicological studies in animal models have reported associations between DBP exposure and pregnancy loss, birth defects, and reduced fetal birth weight [8,9,10]. However, the epidemiologic literature remains equivocal [11,12,13,14].

There is increasing evidence from non-human toxicology studies that DBPs have endocrine disrupting capabilities and the potential to negatively impact the reproductive system [15]. For example, dibromoacetic acid (DBAA) exposure was found to have spermatoxic effects (i.e., delayed spermiation and formation of atypical residual bodies) among male rats and mice [16]. Since other toxicology studies have suggested the possibility of endocrine disruption as a mechanism for hypospadias [17,18,19], there is a need for epidemiologic studies to determine if DBPs are related to the occurrence of this birth defect.

Based on the current epidemiologic literature, it is unclear if maternal DBP exposure increases the risk of hypospadias [20,21,22]. While none of the existing studies reported an association between household DBP concentrations and hypospadias, two case-control studies observed an elevated odds of hypospadias in analyses that incorporated maternal water consumption data to estimate DBP exposure via ingestion [21,22]. Further, the current literature is limited by the fact that none of the existing studies have included data on all nine individual THM and HAA species that are measured under USEPA regulations. Thus, the objective of this study was to evaluate the association between hypospadias and maternal exposure to TTHMs, HAA5s, and individual species of both groups using data from a large population-based, case-control study in the US. In this study, we incorporated individual-level water-use data to account for relevant routes of DBP exposure.

## 2. Materials and Methods

### 2.1. Study Design and Study Population

The National Birth Defects Prevention Study (NBDPS) is a case-control study of birth defects that collected data for deliveries from October 1997 to December 2011 [23]. Ten centers across the US with access to birth defects surveillance systems contributed to the study (Arkansas, California, Georgia, Iowa, Massachusetts, New Jersey, New York, North Carolina, Texas, and Utah). Eligible cases were first identified through these surveillance systems and later verified by a clinical geneticist prior to enrollment in the study. Annually, each center identified approximately 150 eligible unmatched controls (i.e., infants born without a major structural birth defect) within the same catchment area through hospital records and/or birth certificates [24].

Women who agreed to participate in the NBDPS completed a computer-assisted telephone interview approximately 6 weeks to 24 months after the expected date of delivery. During the interview, women provided pregnancy-related information retrospectively on a variety of exposures and lifestyle factors, including usual intake of household drinking water and other water-use, such as bathing. By the end of the study period, the NBDPS collected information on approximately 32,000 cases and 12,000 controls with a response rate of 67% and 65%, respectively [23].

Women were ineligible for the NBDPS if they were incarcerated, participated in the study for a previous pregnancy, did not speak English or Spanish, or did not have legal custody of the child at the time of the interview [23]. To be included in the current analysis, women must have participated in the NBDPS study while the water module was administered as part of the interview (i.e., 2000–2005) and have been residents of one of the eight study areas with available DBP concentration estimates (i.e., California and New Jersey were unable to provide these estimates).

### 2.2. Outcome Classification

The outcomes of interest were second- or third-degree hypospadias (with or without chordee). Therefore, males in the NBDPS with urethral openings located below the coronal region and delivered between 1 January 2000 and 31 December 2005 at one of the eight centers that provided DBP estimates were included as cases. Controls were male infants without a major structural birth defect born in the same study period and study areas as cases.

### 2.3. Exposure Classifications

#### 2.3.1. Overview

**Figure 1:**
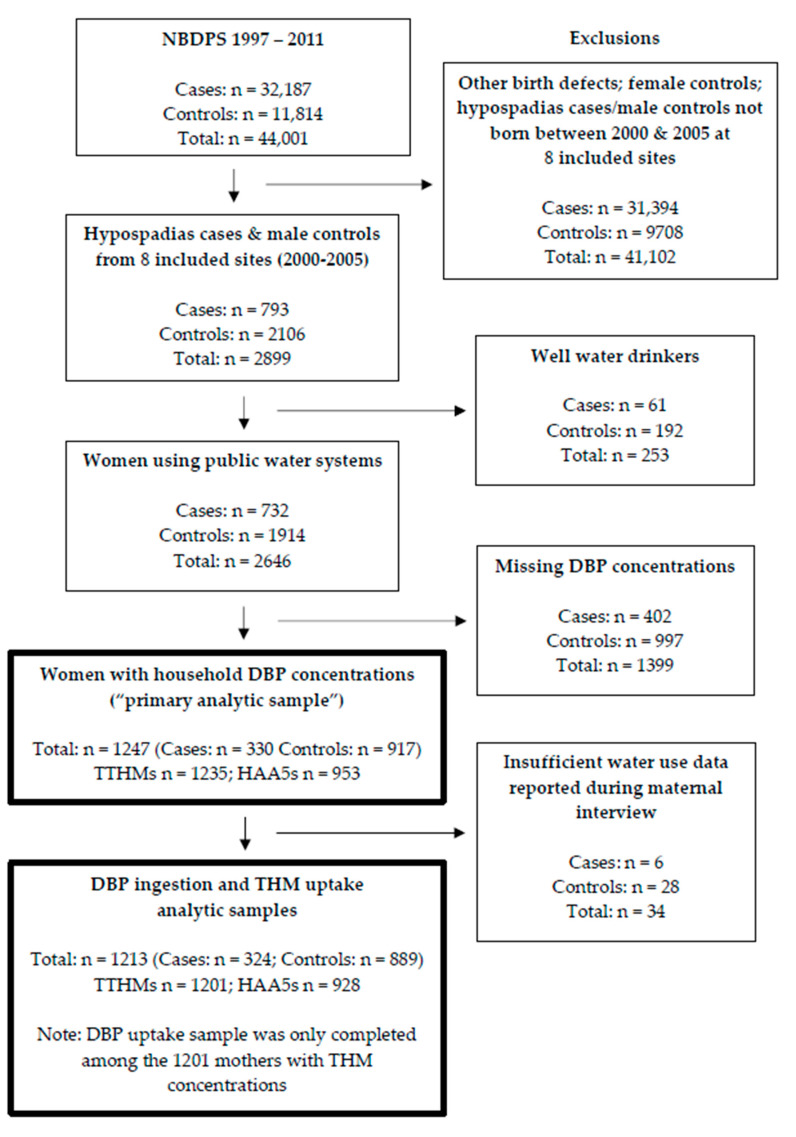
Inclusion and exclusion criteria for each exposure metric.

To comprehensively understand the relationship between DBPs and hypospadias, three different exposure characterizations were considered: (1) household tap water concentrations; (2) ingestion; (3) uptake of THMs via ingestion, showering, and bathing. While the first method only utilized DBP data obtained from PWSs, the other two methods integrated self-reported individual level data on maternal water-use collected using the NBDPS interview. The contaminants of interest were: TTHMs, HAA5s, DBAA, bromoform (BRF), chloroform (CHLF), bromodichloromethane (BDCM), dibromochloromethane (DBCM), monobromoacetic acid (MBAA), monochloroacetic acid (MCAA), dichloroacetic acid (DCAA), and trichloroacetic acid (TCAA). To eliminate the potential for uncontrolled confounding and the effect of other harmful contaminants in well water (e.g., pesticides), women who reported using well water during pregnancy were excluded from all three exposure classifications. The specifics of each exposure characterization are detailed below, and an overview of the study’s inclusion/exclusion criteria is outlined in Figure 1.

#### 2.3.2. DBP Concentrations in Household Tap Water

The collection of DBP data by the NBDPS has been described previously [25], and we summarize the process briefly here. During the interview, women reported their residential history throughout pregnancy and the three months before conception. Because the relevant period of exposure for a majority of structural birth defects is considered to be the month before conception and the first three months of pregnancy (i.e., the periconceptional period) [26], any household addresses reported for this time period were geocoded and linked with a PWS using digital service area maps. In instances where service area maps were not available, 2010 census place shape maps were used to approximate service boundaries. In cities with multiple PWSs that did not clearly define service boundaries, the system serving the largest population was linked with the residence [25].

DBP concentrations for PWSs are made available through the Safe Drinking Water Act [7]. To account for temporal and spatial fluctuation within a single water system, PWSs may take samples from different sites on the same day and multiple times during a calendar year. Thus, for each residence, we (1) calculated the mean DBP concentration for all samples collected on a single day among PWSs that took samples at multiple locations, and (2) estimated inverse time-weighted means if samples were taken on different days during each woman’s periconceptional period, with greater weights applied to measurements taken closer to each woman’s estimated date of conception [25]. If a woman had more than one place of residence during the periconceptional period, time-weighted means were calculated based on the number of days she reported living at each residence.

All women who were successfully linked during this process were included in the analytic sample for this exposure characterization. Women were excluded if the NBDPS was unable to obtain a household THM or HAA concentration (Figure 1). Since these household DBP concentration estimates were also critical for the subsequent exposure characterizations, the women who met the inclusion criteria for this exposure characterization are referred to as the “primary analytic sample”. It is important to note that two study centers, Massachusetts and Utah, did not provide estimates for individual DBP species and were only included in the assessment of TTHMs and HAA5s.

#### 2.3.3. Water Consumption and DBP Ingestion

As part of the water module administered during the NBDPS maternal interview from 2000 through 2005, women provided details about water source(s) (i.e., unfiltered tap, filtered tap, bottled, and other), consumption, and filters at their home(s) and worksite(s). Along with household DBP concentration estimates gathered during the exposure assessment, this information was utilized to estimate a woman’s exposure to DBPs via ingestion. Thus, women missing household concentration estimates or maternal interview information related to water consumption were excluded from this exposure characterization (Figure 1).

Methods developed by the Center for Health Effects of Environmental Contamination (CHEEC) at the University of Iowa were utilized to implement this classification of DBP exposure [25]. If a woman reported no changes in water consumption or source during pregnancy, we multiplied the number of 8 oz. (237 mL) glasses of water she drank from each source by the number of days she was at each home and worksite during the 4-month periconceptional period. To reduce the impact of outliers, we capped the number of glasses drank at home and work to thirty and twenty glasses per day, respectively. We did not include water-used for hot drinks or cooking since consumption amounts were not provided for these types of beverages [25]. We also used the additional information provided by women to reclassify the “other source” responses into one of the three primary sources (i.e., unfiltered tap, filtered tap, or bottled) when possible.

Due to the NBDPS water module structure, two assumptions were applied to women who reported certain changes in water consumption behaviors during pregnancy. The first assumption pertained to women who reported a change in the amount of water they consumed. While women reported the time of this change and how much more or less water they drank, the NBDPS questionnaire did not capture the distribution of this change by water source (i.e., unfiltered tap, filtered tap, bottled). Thus, we utilized an unweighted approach, such that the distribution of sources applied to the change in amount of water consumed was equal to the distribution reported before the change. Secondly, some women reported a change in source during pregnancy; however, the time of this change was not indicated as part of the interview. Therefore, we assumed that any reported changes in source occurred after the periconceptional period. Previous research has shown that both of these assumptions do not substantially impact results when compared to other approaches [25].

After accounting for any reported changes, we multiplied the total number of glasses of water a woman drank from each source by the household DBP concentration estimates. Since worksite addresses were not obtained during the NBDPS interview, we assumed that a woman’s household and worksite(s) were serviced by the same PWS. For filters known to remove DBPs, we applied a 90% reduction to the reported DBP concentration [25]. A 10% reduction was used for filters unable to remove DBPs or with an unknown capability of removing DBPs. Bottled water was assumed to contribute 0 μg/L to DBP exposure [25]. We summed exposure from all sources and then divided by 120 days to obtain a woman’s average daily ingestion of DBPs (μg/day) during the periconceptional period.

#### 2.3.4. Uptake of THMs via Ingestion, Showering, and Bathing

Due to the volatile nature of THMs (but not HAAs), we considered a third exposure measure that accounted for potential THM exposure from showering and/or bathing. Based on information provided during the maternal interview, we calculated the average daily duration of these behaviors for each woman. The weekly number of showers and baths was capped to 30 to reduce the potential impact of outliers. We multiplied these values by household THM concentrations and uptake factors of 0.001538 and 0.001312 μg of THMs in blood per minute per microgram from showering and bathing, respectively [12]. DBP ingestion estimates were multiplied by an uptake factor of 0.00490 and merged with showering and bathing estimates to obtain an integrated index of blood concentration [12].

### 2.4. Analysis

We first developed a directed acyclic graph (DAG) [27] to a priori identify factors associated with DBPs and hypospadias. Based on the current literature, we determined that study center, maternal age at conception (<20, 20–25, 26–35, 36+ years), maternal race/ethnicity (non-Hispanic white, non-Hispanic black, Hispanic, other), pre-pregnancy body mass index (BMI, kg/m^2^) (underweight (<18.5); normal weight (18.5–24.9); overweight (25.0–29.9); obese (≥30.0)), maternal education (<high school, high school, >high school), and parity (0, 1, ≥2 previous live births) were all potential confounders in this analysis. To understand the demographic characteristics of our primary analytic sample, we evaluated the distribution of these covariates by case status. Since there was sample attrition after excluding women who were not successfully linked with a PWS and women who reported drinking well water, we used Fisher’s exact tests to determine if there were any differences among women included in our primary analytic sample and women excluded for one of these two reasons. We used Fisher’s exact tests to account for the potentially small sample sizes of some groups (i.e., *n* ≤ 5).

To account for the case-control study design, we used multivariable logistic regression to estimate adjusted odds ratios and 95% confidence intervals to evaluate the association between maternal DBP exposure and hypospadias. For all three exposure characterizations, we used three categories to categorize the magnitude of maternal DBP exposure based on the distribution of exposure among controls: (Q1) <50%, (Q2) ≥ 50% to < 75%, and (Q3) ≥ 75%. However, if more than one-half of the controls had no exposure, all unexposed women were included in the lowest quantile (Q1). In each of these models, Q1 was used as the referent category. We also completed a secondary analysis using the USEPA’s maximum contaminant levels (MCL) for TTHMs and HAA5s to create a binary categorization (≤MCL, >MCL) exclusively for household tap water concentrations. Lastly, we repeated the analysis of household tap water concentrations after restricting to women with complete water-use data to confirm no bias was induced due to missing water-use data from the interview. All analyses were completed in SAS 9.4 (SAS Institute, Inc; Cary, NC, USA) using complete case analysis methods.

## 3. Results

After excluding women who drank well water (*n* = 253) and women missing DBP concentrations (*n* = 1399), a total of 1247 women (330 cases; 917 controls) remained in our primary analytic sample to evaluate the association between household DBP concentrations and hypospadias (Figure 1). The maternal characteristics of this sample are presented in Table 1. Factors such as maternal age at conception, pre-pregnancy BMI, and family history of hypospadias were similar between cases and controls. A greater proportion of cases than controls were nulliparous (0 previous live births: 54.9% vs. 42.0%), non-Hispanic white (71.5% vs. 58.9%), and more highly educated (>high school: 76.4% vs. 60.3%). Although the study site distributions were generally comparable by case status, there were notable differences in the relative contribution of cases and controls from Texas and Massachusetts.

Differences were found between included cases and those excluded for drinking well water by maternal education (*p* = 0.04) and study site (*p* < 0.001) (Table S1). Controls in these two study sample categories differed by maternal race/ethnicity (*p* < 0.001), the number of previous live births (*p* = 0.008), and study site (*p* <0.001). When we compared cases in our primary analytic sample to cases that were excluded for missing DBP concentrations, we observed differences by study site (*p* < 0.001). Controls differed by maternal race/ethnicity (*p* = 0.04), pre-pregnancy BMI (*p* = 0.005), and study site (*p* < 0.001) (Table S2).

### 3.1. Household Tap Water Concentrations and Hypospadias

In the assessment of household tap water concentrations, null associations were observed with increasing TTHM or HAA5 exposure (aOR range: 0.8 to 1.1) (Table 2). Moreover, null associations were detected for TTHM and HAA5 exposure categorized according to the USEPA’s MCL values (Table 3). An increased risk of hypospadias was observed among women with household BDCM levels in the second quantile (aOR: 1.8; 95% CI: 1.2, 2.7; Table 2). Strong inverse associations were observed for women in the highest quantiles of BRF (aOR: 0.3; 95% CI: 0.1, 0.8) and DBCM (aOR: 0.5; 95% CI: 0.3, 0.9) exposure. All results were relatively unchanged after restricting the analyses to women with complete water-use data (Tables S3 and S4).

### 3.2. DBP Ingestion and Hypospadias

A total of 34 women in our primary analytic sample did not report sufficient information about water-use during the maternal interview and were excluded from analyses evaluating DBP ingestion and hypospadias (*n* = 1213). The results for this exposure metric are reported in Table 4. No excess risk was observed with increasing DBP exposure due to ingestion (aOR range: 0.5 to 1.0).

### 3.3. Uptake of THMs via Ingestion, Showering, and Bathing

A total of 1201 women were included in the analysis of THM uptake by ingestion, showering, and bathing and hypospadias (Table 5). With the exception of the second quantile of BDCM exposure, adjusted odds ratios in this analysis tended to be equal to or below 1.0 (aOR range: 0.5 to 1.3).

## 4. Discussion

Findings from the present analysis do not support the hypothesis that maternal DBP exposure is associated with 2nd and 3rd degree hypospadias. In our study, an increased risk of hypospadias was observed among infants of women with household BDCM concentrations in the second quantile. However, there was no evidence of a monotonic dose–response relationship and this association did not persist after the inclusion of individual-level behavior data. Our results support the majority of existing epidemiologic evidence that maternal DBP exposure is not associated with hypospadias.

Three studies have evaluated the relationship between DBPs and hypospadias [20,21,22]. One cross-sectional study utilized information from Taiwan’s birth and “Waterworks” registries (*n* = 72 hypospadias cases) [20]. The study did not observe an association between TTHM exposure and hypospadias, but it is important to note that this study did not include water-use, HAA, or individual THM species data. Two case-control studies were able to incorporate both water quality and maternal behavior data to estimate the risk of hypospadias using several exposure metrics [21,22]. Izatt et al. focused on THM exposure and water-use behaviors in England (cases: *n* = 471; controls: *n* = 490). Luben et al. analyzed NBDPS participants from Arkansas with deliveries between 2000 and 2002 (40 cases and 242 controls). This early NBDPS study included data on individual HAA species but lacked information on individual THM species. In both of these studies, no association was observed between tap water DBP concentrations and hypospadias. However, Izatt et al. observed an increased risk of hypospadias among women in the highest exposure category of BDCM ingestion and Luben et al. noted an increased risk of hypospadias for the intermediate tertile of TTHM exposure due to ingestion. Our results, based on a large multi-site study, provide additional epidemiologic evidence that DBPs present in tap water are not likely to negatively impact male urethral development during gestation. However, given that Izatt and colleagues’ outcome classification is unclear (i.e., may include mild hypospadias), Hwang and colleagues used a cross-sectional study design, and our study used different exposure category cut-points, it is difficult to draw direct comparisons between our results and those of the previous studies.

Environmental exposures with endocrine disrupting capabilities are hypothesized to play a role in the occurrence of hypospadias [28,29]. Given that hormones are critical in the masculinization of the external genitalia during male fetal development [30], it is hypothesized that exogenous exposures that interfere with androgen or estrogen pathways may partially explain malformations of the male urethra [31,32]. Several animal models support this notion and have demonstrated that exposure to endocrine disrupting chemicals can induce hypospadias in experimental settings [17,18,19]. Recent studies have shown that some regulated DBPs have endocrine-disrupting properties [33,34], providing biological plausibility for the relationship between hypospadias and DBPs. However, since inconsistent results have been observed in the epidemiologic studies of hypospadias among well-known endocrine disruptors such as pesticides [26,35,36], and the etiology of hypospadias remains relatively unknown, additional epidemiologic research is needed to understand the potential contribution of endocrine-disrupting chemicals, including DBPs, to this multifactorial birth defect [15,29].

We also cannot ignore the potential impact of exposure misclassification on our results. PWSs in the US normally calculate long-running annual averages (LRAAs) based on quarterly data from a limited number of testing sites throughout a distribution system. PWSs that serve small populations (i.e., <10,000 residents) collect samples even less frequently and are only required to conduct water quality assessments on an annual basis [7]. Given the temporal and spatial variability of DBPs throughout a distribution system [37,38], we cannot rule out that such sparse water sampling requirements contribute to exposure misclassification in our study. This is an important consideration due to the relatively short window of exposure relevant to birth defects and that LRAAs and quarterly averages can be problematic when used for brief exposure periods [39]. In addition to this Berkson-type measurement error, we must also consider that the DBP concentration estimates utilized in this study are susceptible to classical measurement error [40]. Lastly, in this study we made several assumptions about drinking water habits, sources, and filtration capabilities. While several of these assumptions pertained to a small number of women, such as the near-complete removal of DBPs via select filters (<1% of women), we recognize that there is a potential for exposure measurement error. Although we do not believe that any of these sources of measurement error would be differential by case-status, the various sources of exposure measurement error may, on average, have diluted associations.

Individuals that rely on private wells have been included in other epidemiologic studies related to DBP exposure [41,42]. They are typically assumed to have no exposure to these contaminants because of the limited use of disinfectant treatments reported among this population [25]. However, we chose not to include women who drank well water in our study for two reasons. First, we were concerned that well-water drinkers may be different from the rest of our study sample (race/ethnicity, education, etc.). Generally, these individuals comprise part of the low-exposure category due to the assumption that individuals who drink well water are unexposed to DBPs, but we did not think it was appropriate to include this population in comparisons with women who rely on PWSs. This was confirmed by the various differences detected in our Fisher’s exact tests between women who drank well water and our primary analytic sample. We presume that the differences we observed in our study are related to residential status (i.e., rural vs. urban/suburban) since they are generally consistent with previously reported differences between urban and rural women [43]. Second, we wanted to eliminate the potentially harmful effects of other exposures found with relatively higher concentrations in well water, such as arsenic and nitrates, that could possibly distort our results [44,45]. We suggest that future studies on DBPs and adverse reproductive outcomes consider these issues.

There are other limitations in our study that need to be considered. First, we could not obtain DBP concentrations for all of our eligible cases and controls, and we noted differences in demographic and other factors between women with and without exposure assignments, which indicates a potential for selection bias to affect our results if these factors are related to both DBP exposure potential and hypospadias risk. The retrospective design of the NBDPS allows for potential recall error since women were asked to report on water-use behaviors during the periconceptional period up to 24 months after delivery. However, we would assume that any misclassification due to recall would be nondifferential by case status with results expected to be biased toward the null. Lastly, although we accounted for all confounders identified in our DAG, there is still a potential for uncontrolled confounding, even by unregulated DBPs (e.g., haloacetonitriles and haloacetaldehydes) or other non-DBP water contaminants, given the limited amount of established epidemiologic information on hypospadias currently available. Unmeasured potential confounders could bias effect estimates toward or away from the null. We do not have the necessary information to estimate the magnitude of bias resulting from unmeasured potential confounders.

Our study has several strengths. First, we completed a comprehensive assessment of this specific exposure–outcome relationship by including all individual THM and HAA species that fall under USEPA regulations. This is important because in limiting the analyses to aggregate DBP groups (i.e., TTHMs and HAA5s), studies may not be able to determine if specific DBP species are more harmful than others, and they may be masking key associations. Another strength of this study is that we used multiple exposure metrics, which is critical given that volatile DBPs can be ingested, absorbed, and inhaled [46]. Lastly, by building upon the study completed by Luben and colleagues [22] and leveraging the large size of the NBDPS, we were able to complete the largest epidemiologic evaluation of DBPs and hypospadias in the United States.

## 5. Conclusions

By combining data from PWSs and maternal water-use information from the NBDPS maternal interview, we were able to estimate the association between DBPs and hypospadias using various metrics. In the context of exposure during pregnancy, our results are consistent with existing evidence that current DBP levels in tap water are not strongly associated with an increased risk of hypospadias. The results of our study suggest that the USEPA’s current MCLs for DBPs are sufficient with regard to this specific birth defect. However, due to the pervasiveness of DBPs in US water systems and their endocrine disrupting potential, it is prudent that these contaminants continue to be monitored and evaluated in relation to adverse reproductive outcomes.

## Figures and Tables

**Table 1:** Distribution of maternal characteristics among hypospadias cases and controls, National Birth Defects Prevention Study, 2000–2005 (*n* = 1247).

Maternal Characteristic	Cases ^α^		Controls	
	*n*	%	*n*	%
Maternal age at conception				
<20 years	26	7.9	110	12.0
20–25 years	81	24.6	264	28.8
26–35 years	182	55.2	468	51.0
36+ years	41	12.4	75	8.2
Maternal race/ethnicity				
Non-Hispanic White	236	71.5	540	58.9
Non-Hispanic Black	49	14.9	136	14.8
Hispanic	20	6.1	175	19.1
Other	25	7.6	66	7.2
Pre-pregnancy body mass index *				
Underweight	19	5.9	40	4.6
Normal weight	160	49.5	462	52.6
Overweight	86	26.6	210	23.9
Obese	58	18.0	166	18.9
Missing	7		39	
Maternal education				
<High school	25	7.6	145	15.8
High school	53	16.1	219	23.9
>High school	252	76.4	552	60.3
Missing	0		1	
Number of previous livebirths				
0	181	54.9	385	42.0
1	97	29.4	290	31.6
≥2	52	15.8	242	26.4
Family history of hypospadias (1 degree relative)				
No	319	96.7	913	99.6
Yes	11	3.3	4	0.4
Study Site				
Arkansas	62	18.8	157	17.1
Georgia	64	19.4	164	17.9
Iowa	28	8.5	142	15.5
Massachusetts	97	29.4	128	14.0
New York	5	1.5	27	2.9
North Carolina	46	13.9	128	14.0
Texas	6	1.8	127	13.9
Utah	22	6.7	44	4.8

* Underweight (<18.5); Normal weight (18.5–24.9); Overweight (25–29.9); Obese (≥30). ^α^ 293 cases were isolated; 37 were multiples.

**Table 2:** Association between household disinfection by-product (DBP) concentrations and hypospadias, National Birth Defects Prevention Study, 2000–2005 (*n* = 1247) *.

DBP	Quantile	μg/L	Total	Cases	Controls	OR	95% CI	aOR ^φ^	95% CI
**TTHM**			**1235**						
	Q1 (<50%)	<37.2		162 (50.0)	455 (50.0)	1.0	REF	1.0	REF
	Q2 (≥50%–<75%)	≥37.2–<52.9		89 (27.5)	228 (25.0)	1.1	0.8, 1.5	1.1	0.8, 1.5
	Q3 (≥75%)	≥52.9		73 (22.5)	228 (25.0)	0.9	0.7, 1.2	0.8	0.5, 1.1
**BRF**			**869**						
	Q1 (<50%)	<0.5		121 (63.4)	339 (50.0)	1.0	REF	1.0	REF
	Q2 (≥50%–<75%)	≥0.5–<3.1		52 (27.2)	169 (24.9)	0.9	0.6, 1.3	0.6	0.3, 1.2
	Q3 (≥75%)	≥3.1		18 (9.4)	170 (25.1)	0.3	0.2, 0.5	0.3	0.1, 0.8
**CHLF**			**869**						
	Q1 (<50%)	<19.7		66 (34.6)	339 (50.0)	1.0	REF	1.0	REF
	Q2 (≥50%–<75%)	≥19.7–<35.0		64 (33.5)	168 (24.8)	2.0	1.3, 2.9	1.2	0.8, 1.9
	Q3 (≥75%)	≥35.0		61 (31.9)	171 (25.2)	1.8	1.2, 2.7	1.1	0.7, 1.8
**BDCM**			**868**						
	Q1 (<50%)	<7.0		82 (42.9)	338 (49.9)	1.0	REF	1.0	REF
	Q2 (≥50%–<75%)	≥7.0–<11.0		72 (37.7)	167 (24.7)	1.8	1.2, 2.6	1.8	1.2, 2.7
	Q3 (≥75%)	≥11.0		37 (19.4)	172 (25.4)	0.9	0.6, 1.4	0.7	0.4, 1.1
**DBCM**			**867**						
	Q1 (<50%)	<2.5		127 (66.8)	335 (49.5)	1.0	REF	1.0	REF
	Q2 (≥50%–<75%)	≥2.5–<7.0		44 (23.2)	173 (25.6)	0.7	0.5, 1.0	0.8	0.5, 1.3
	Q3 (≥75%)	≥7.0		19 (10.0)	169 (25.0)	0.3	0.2, 0.5	0.5	0.3, 0.9
**HAA5**			**953**						
	Q1 (<50%)	<24.5		148 (53.8)	335 (49.4)	1.0	REF	1.0	REF
	Q2 (≥50%–<75%)	≥24.5–<37.4		61 (22.2)	173 (25.5)	0.8	0.6, 1.1	0.8	0.6, 1.2
	Q3 (≥75%)	≥37.4		66 (24.0)	170 (25.1)	0.9	0.6, 1.2	0.8	0.6, 1.2
**MBAA**			**759**						
	Q1 (No exposure) ^α^	0		112 (58.0)	325 (57.4)	1.0	REF	1.0	REF
	Q2 (<75%)	>0–<1.0		16 (8.3)	38 (6.8)	1.2	0.7, 2.3	1.5	0.7, 3.0
	Q3 (≥75%)	≥1.0		65 (33.7)	203 (35.9)	0.9	0.7, 1.3	0.8	0.5, 1.4
**MCAA**			**759**						
	Q1 (<50%)	<1.3		103 (53.4)	283 (50.0)	1.0	REF	1.0	REF
	Q2 (≥50%–<75%)	≥1.3–<3.5		48 (24.9)	141 (24.9)	0.9	0.6, 1.4	0.7	0.4, 1.3
	Q3 (≥ 75%)	≥ 3.5		42 (21.8)	142 (25.1)	0.8	0.5, 1.2	0.6	0.4, 1.2
**DBAA**			**759**						
	Q1 (<50%)	<0.9		113 (58.6)	283 (50.0)	1.0	REF	1.0	REF
	Q2 (≥50%–<75%)	≥0.9–<2.1		55 (28.5)	141 (24.9)	1.0	0.7, 1.4	1.0	0.6, 1.6
	Q3 (≥75%)	≥2.1		25 (13.0)	142 (25.1)	0.4	0.3, 0.7	0.6	0.3, 1.1
**DCAA**			**759**						
	Q1 (<50%)	<12.4		91 (47.15)	283 (50.0)	1.0	REF	1.0	REF
	Q2 (≥50%–<75%)	≥12.4–<19.9		50 (25.9)	141 (24.9)	1.1	0.7, 1.6	0.8	0.5, 1.3
	Q3 (≥75%)	≥ 19.9		52 (26.9)	142 (25.1)	1.1	0.8, 1.7	0.8	0.5, 1.3
**TCAA**			**759**						
	Q1 (<50%)	<9.4		88 (45.6)	285 (50.4)	1.0	REF	1.0	REF
	Q2 (≥50%–<75%)	≥9.4–<15.8		46 (23.8)	139 (24.6)	1.1	0.7, 1.6	0.8	0.5, 1.3
	Q3 (≥75%)	≥15.8		59 (30.6)	142 (25.1)	1.3	0.9, 2.0	1.0	0.6, 1.5

* Massachusetts and Utah did not provide exposure estimates for individual DBP species; ^α^ Due to low concentration estimates, referent includes all unexposed women (i.e., concentration = 0 μg/L); ^φ^ Adjusted for: maternal age at conception, study site, parity, maternal education, pre-pregnancy body mass index, and maternal race/ethnicity; Total Trihalomethanes (TTHM); Bromoform (BRF); Chloroform (CHLF); Bromodichloromethane (BDCM); Dibromochloromethane (DBCM); Total Haloacetic Acids (HAA5); Monobromoacetic Acid (MBAA); Monochloroacetic Acid (MCAA); Dibromoacetic Acid (DBAA); Dichloroacetic Acid (DCAA); Trichloroacetic Acid (TCAA).

**Table 3:** Association between household disinfection by-product (DBP) concentrations above and below US Environmental Protection Agency (USEPA) allowable levels and hypospadias, National Birth Defects Prevention Study, 2000–2005 (*n* = 1247).

Regulation Categorizations	Cases	Controls	OR	95% CI	aOR *	95% CI
**Total Trihalomethanes** (TTHMs) ≤ 80 μg/L	300 (92.6)	834 (91.6)	1.0	REF	1.0	REF
TTHMs > 80 μg/L	24 (7.4)	77 (8.5)	0.9	0.5, 1.4	0.7	0.4, 1.1
Total	324	911				
**Total Haloacetic Acids** (HAA5s) ≤ 60 μg/L	257 (93.5)	623 (91.9)	1.0	REF	1.0	REF
HAA5s > 60 μg/L	18 (6.6)	55 (8.1)	0.8	0.5, 1.4	0.8	0.4, 1.4
Total	275	678				

* Adjusted for: maternal age at conception, study site, parity, maternal education, pre-pregnancy body mass index, and maternal race/ethnicity.

**Table 4:** Association between maternal ingestion of disinfection by-products (DBPs) and hypospadias, National Birth Defects Prevention Study, 2000–2005 (*n* = 1213) *.

DBP	Quantile	μg/Day	Total	Cases	Controls	OR	95% CI	aOR ^φ^	95% CI
**TTHM**			**1201**						
	Q1 (<50%)	<16.3		163 (51.3)	442 (50.1)	1.0	REF	1.0	REF
	Q2 (≥50%–<5%)	≥16.3–<49.6		85 (26.7)	222 (25.1)	1.0	0.8, 1.4	1.0	0.7, 1.4
	Q3 (≥75%)	≥49.6		70 (22.0)	219 (24.8)	0.9	0.6, 1.2	0.8	0.5, 1.1
**BRF**			**842**						
	Q1 (No exposure) ^α^	0		127 (67.9)	393 (60.0)	1.0	REF	1.0	REF
	Q2 (<75%)	>0–<1.2		25 (13.4)	99 (15.1)	0.8	0.5, 1.3	0.6	0.4, 1.2
	Q3 (≥75%)	≥1.2		35 (18.7)	163 (24.9)	0.7	0.4, 1.0	0.7	0.4, 1.2
**CHLF**			**842**						
	Q1 (<50%)	<7.5		76 (40.6)	327 (49.9)	1.0	REF	1.0	REF
	Q2 (≥50%–<75%)	≥7.5–<28.5		58 (31.0)	164 (25.0)	1.5	1.0, 2.2	1.0	0.6, 1.5
	Q3 (≥75%)	≥28.5		53 (28.3)	164 (25.0)	1.4	0.9, 2.1	0.8	0.5, 1.3
**BDCM**			**841**						
	Q1 (<50%)	<3.6		88 (47.1)	327 (50.0)	1.0	REF	1.0	REF
	Q2 (≥50%–<75%)	≥3.6–<10.7		52 (27.8)	163 (24.9)	1.2	0.8, 1.8	0.9	0.6, 1.5
	Q3 (≥75%)	≥10.7		47 (25.1)	164 (25.1)	1.1	0.7, 1.6	0.9	0.6, 1.4
**DBCM**			**840**						
	Q1 (<50%)	<1.1		109 (58.6)	327 (50.0)	1.0	REF	1.0	REF
	Q2 (≥50%–<75%)	≥1.1–<4.1		45 (24.2)	164 (25.1)	0.8	0.6, 1.2	0.7	0.4, 1.0
	Q3 (≥75%)	≥4.1		32 (17.2)	163 (24.9)	0.6	0.4, 0.9	0.7	0.4, 1.1
**HAA5**			**928**						
	Q1 (<50%)	<11.5		151 (55.7)	328 (49.9)	1.0	REF	1.0	REF
	Q2 (≥ 50%–<75%)	≥11.5–<33.7		66 (24.4)	164 (25.0)	0.9	0.6, 1.2	0.9	0.6, 1.3
	Q3 (≥75%)	≥33.7		54 (19.9)	165 (25.1)	0.7	0.5, 1.0	0.7	0.5, 1.1
**MBAA**			**736**						
	Q1 (No exposure) ^α^	0		134 (70.5)	360 (65.9)	1.0	REF	1.0	REF
	Q2 (<75%)	>0–<0.7		16 (8.4)	50 (9.2)	0.9	0.5, 1.6	0.7	0.4, 1.4
	Q3 (≥75%)	≥0.7		40 (21.1)	136 (24.9)	0.8	0.5, 1.2	0.7	0.4, 1.1
**MCAA**			**736**						
	Q1 (No exposure) ^α^	0		116 (61.1)	298 (54.6)	1.0	REF	1.0	REF
	Q2 (<75%)	>0–<2.5		29 (15.3)	112 (20.5)	0.7	0.4, 1.1	0.5	0.3, 0.9
	Q3 (≥75%)	≥2.5		45 (23.7)	136 (24.9)	0.9	0.6, 1.3	0.6	0.4, 1.0
**DBAA**			**736**						
	Q1 (No exposure) ^α^	0		123 (64.7)	326 (59.7)	1.0	REF	1.0	REF
	Q2 (<75%)	>0–<1.2		26 (13.7)	83 (15.2)	0.8	0.5, 1.4	0.7	0.4, 1.2
	Q3 (≥75%)	≥1.2		41 (21.6)	137 (25.1)	0.8	0.5, 1.2	0.8	0.5, 1.3
**DCAA**			**736**						
	Q1 (<50%)	<6.5		90 (47.4)	273 (50.0)	1.0	REF	1.0	REF
	Q2 (≥50%–<75%)	≥6.5–<17.8		57 (30.0)	136 (24.9)	1.3	0.9, 1.9	1.0	0.6, 1.5
	Q3 (≥75%)	≥17.8		43 (22.6)	137 (25.1)	1.0	0.6, 1.4	0.7	0.5, 1.2
**TCAA**			**736**						
	Q1 (<50%)	<3.6		92 (48.4)	273 (50.0)	1.0	REF	1.0	REF
	Q2 (≥50%–<75%)	≥3.6–<13.8		52 (27.4)	137 (25.1)	1.1	0.8, 1.7	0.8	0.5, 1.3
	Q3 (≥75%)	≥13.8		46 (24.2)	136 (24.9)	1.0	0.7, 1.5	0.7	0.5, 1.2

* Massachusetts and Utah did not provide exposure estimates for individual DBP species. ^α^ Due to low exposure estimates, referent includes all unexposed women (i.e., exposure = 0 μg/day). ^φ^ Adjusted for: maternal age, study site, parity, maternal education, pre-pregnancy body mass index, and maternal race/ethnicity. Total Trihalomethanes (TTHM); Bromoform (BRF); Chloroform (CHLF); Bromodichloromethane (BDCM); Dibromochloromethane (DBCM); Total Haloacetic Acids (HAA5); Monobromoacetic Acid (MBAA); Monochloroacetic Acid (MCAA); Dibromoacetic Acid (DBAA); Dichloroacetic Acid (DCAA); Trichloroacetic Acid (TCAA).

**Table 5:** Association between hypospadias and maternal uptake of disinfection by-product (DBP) by ingestion/showering/bathing, National Birth Defects Prevention Study, 2000–2005 (*n* = 1201) *.

DBP	Quantile	μg/Day	Total	Cases	Controls	OR	95% CI	aOR ^φ^	95% CI
**TTHM**			**1201**						
	Q1 (<50%)	<2.60		175 (55.0)	441 (49.9)	1.0	REF	1.0	REF
	Q2 (≥50%–<75%)	≥2.60–<4.60		81 (25.5)	221 (25.0)	0.9	0.7,1.3	1.0	0.7, 1.4
	Q3 (≥75%)	≥4.60		62 (19.5)	221 (25.0)	0.7	0.5, 1.0	0.7	0.5, 1.0
**BRF**			**842**						
	Q1 (<50%)	<0.04		116 (62.0)	327 (49.9)	1.0	REF	1.0	REF
	Q2 (≥50%–<75%)	≥0.04–<0.26		51 (27.3)	164 (25.0)	0.9	0.6, 1.3	0.8	0.4, 1.5
	Q3 (≥75%)	≥0.26		20 (10.7)	164 (25.0)	0.3	0.2, 0.6	0.5	0.2, 1.1
**CHLF**			**842**						
	Q1 (<50%)	<1.43		78 (41.7)	327 (49.9)	1.0	REF	1.0	REF
	Q2 (≥50%–<75%)	≥1.43–<2.99		51 (27.3)	164 (25.0)	1.3	0.9, 1.9	0.7	0.5, 1.2
	Q3 (≥75%)	≥2.99		58 (31.0)	164 (25.0)	1.5	1.0, 2.2	0.8	0.5, 1.3
**BDCM**			**841**						
	Q1 (<50%)	<0.54		89 (47.6)	327 (50.0)	1.0	REF	1.0	REF
	Q2 (≥50%–<75%)	≥0.54–<1.00		60 (32.1)	163 (24.9)	1.4	0.9, 2.0	1.3	0.8, 1.9
	Q3 (≥75%)	≥1.00		38 (20.3)	164 (25.1)	0.9	0.6, 1.3	0.7	0.5, 1.2
**DBCM**			**840**						
	Q1 (<50%)	<0.33		107 (57.5)	327 (50.0)	1.0	REF	1.0	REF
	Q2 (≥50%–<75%)	≥0.33–<0.70		43 (23.1)	163 (24.9)	0.8	0.5, 1.2	1.0	0.7, 1.6
	Q3 (≥75%)	≥0.70		36 (19.4)	164 (25.1)	0.7	0.4, 1.0	0.9	0.6, 1.5

* Massachusetts and Utah did not provide exposure estimates for individual DBP species. ^φ^ Adjusted for: maternal age at conception, study site, parity, maternal education, pre-pregnancy body mass index, and maternal race/ethnicity. Total Trihalomethanes (TTHM); Bromoform (BRF); Chloroform (CHLF); Bromodichloromethane (BDCM); Dibromochloromethane (DBCM).

## Supplementary Materials

The following are available online at https://www.mdpi.com/1660-4601/17/24/9564/s1, **Table S1**. Comparison of maternal characteristics between primary analytic sample and well water drinkers, NBDPS (2000–2005); Table S2. Comparison of maternal characteristics between primary analytic sample and individuals excluded for missing DBP measurements, National Birth Defects Prevention Study (2000–2005); Table S3. Association between household DBP concentrations and hypospadias restricted to mothers with complete water-use data, National Birth Defects Prevention Study 2000–2005 (*n* = 1213); Table S4. Association between household disinfection by-product (DBP) concentrations above and below US Environmental Protection Agency (EPA) allowable levels and hypospadias restricted to mothers with complete water-use data, National Birth Defects Prevention Study 2000–2005 (*n* = 1213).
