# Supplementary material for: Maternal Exposure to Disinfection By-Products and Risk of Hypospadias in the National Birth Defects Prevention Study (2000–2005)
Source: Int J Environ Res Public Health. 2020 Dec 21;17(24):9564. doi: 10.3390/ijerph17249564 (PMC7766973; doi:10.3390/ijerph17249564)
Supplement: Supplementary file 1 [file ijerph-17-09564-s001.pdf]

Table S1. Comparison of maternal characteristics between primary analytic sample and well water drinkers, NBDPS (2000-2005)

	**Cases**			**Controls**		
	Primary analytic sample	Well water drinkers	Fisher's exact test	Primary analytic sample	Well water drinkers	Fisher's exact test
**Maternal characteristic**	*n (%)*	*n (%)*	*P-Value*	*n (%)*	*n (%)*	*P-Value*
Maternal age at conception			0.93			0.08
<20 years	26 (7.9)	4 (6.6)		110 (12.0)	21 (10.9)	
20-25 years	81 (24.6)	17 (27.9)		264 (28.8)	47 (24.5)	
26-35 years	182 (55.2)	34 (55.7)		468 (51.0)	97 (50.5)	
36+ years	41 (12.4)	6 (9.8)		75 (8.2)	27 (14.1)	
Maternal race/ethnicity			0.42			<0.001
Non-Hispanic White	236 (71.5)	50 (82.0)		540 (58.9)	139 (72.4)	
Non-Hispanic Black	49 (14.9)	5 (8.2)		136 (14.8)	9 (4.7)	
Hispanic	20 (6.1)	2 (3.3)		175 (19.1)	27 (14.1)	
Other	25 (7.6)	4 (6.6)		66 (7.2)	17 (8.9)	
Pre-pregnancy body mass index			0.38			0.68
Underweight (<18.5)	19 (5.9)	4 (6.6)		40 (4.6)	10 (5.5)	
Normal weight (18.5 – 24.9)	160 (49.5)	35 (57.4)		462 (52.6)	88 (48.4)	
Overweight (25–29.9)	86 (26.6)	10 (16.4)		210 (23.9)	45 (24.7)	
Obese (≥30)	58 (18.0)	12 (19.7)		166 (18.9)	39 (21.4)	
Missing	7			39	10	
Maternal education			0.04			0.30
< High school	25 (7.6)	5 (8.2)		145 (15.8)	32 (16.7)	
High school	53 (16.1)	18 (29.5)		219 (23.9)	55 (28.7)	
> High school	252 (76.4)	38 (62.3)		552 (60.3)	105 (54.7)	
Missing*				1		
Number of previous livebirths			0.67			0.008
0	181 (54.9)	30 (49.2)		385 (42.0)	64 (33.3)	
1	97 (29.4)	21 (34.4)		290 (31.6)	56 (29.2)	
≥2	52 (15.8)	10 (16.4)		242 (26.4)	72 (37.5)	
Family history of hypospadias			1.00			1.00
No	319 (96.7)	59 (96.7)		913 (99.6)	192 (100.0)	
Yes	11 (3.3)	2 (3.3)		4 (0.4)	0 (0.0)	

Table S1 (Cont). Comparison of maternal characteristics between primary analytic sample and well water drinkers, NBDPS (2000-2005)

	Cases			Controls		
	Primary analytic sample	Well water drinkers	Fisher's exact test	Primary analytic sample	Well water drinkers	Fisher's exact test
**Maternal characteristic**	*n (%)*	*n (%)*	*P-Value*	*n (%)*	*n (%)*	*P-Value*
Study Site			<0.001			<0.001
Arkansas	62 (18.8)	9 (14.8)		157 (17.1)	24 (12.5)	
Georgia	64 (19.4)	3 (4.9)		164 (17.9)	9 (4.7)	
Iowa	28 (8.5)	5 (8.2)		142 (15.5)	30 (15.6)	
Massachusetts	97 (29.4)	11 (18.0)		128 (14.0)	24 (12.5)	
New York	5 (1.5)	11 (18.0)		27 (2.9)	36 (18.8)	
North Carolina	46 (13.9)	19 (31.2)		128 (14.0)	33 (17.2)	
Texas	6 (1.8)	2 (3.3)		127 (13.9)	24 (12.5)	
Utah	22 (6.7)	1 (1.6)		44 (4.8)	12 (6.3)	
Total	330	61		917	192	

*Missing values were not evaluated in Fisher's Exact

Table S2. Comparison of maternal characteristics between primary analytic sample and individuals excluded for missing DBP measurements, National Birth Defects Prevention Study (2000-2005)

	Cases			Controls		
	Primary analytic sample	Missing DBP measurements	Fisher's exact test	Primary analytic sample	Missing DBP measurements	Fisher's exact test
**Maternal characteristic**	*n (%)*	*n (%)*	*P-Value*	*n (%)*	*n (%)*	*P-Value*
Maternal age at conception			0.52			0.30
<20 years	26 (7.9)	24 (6.0)		110 (12.0)	145 (14.5)	
20-25 years	81 (24.6)	90 (22.4)		264 (28.8)	298 (29.9)	
26-35 years	182 (55.2)	228 (56.7)		468 (51.0)	475 (47.6)	
36+ years	41 (12.4)	60 (14.9)		75 (8.2)	79 (7.9)	
Maternal race/ethnicity			0.21			0.04
Non-Hispanic White	236 (71.5)	295 (73.4)		540 (58.9)	644 (64.6)	
Non-Hispanic Black	49 (14.9)	64 (15.9)		136 (14.8)	115 (11.5)	
Hispanic	20 (6.1)	27 (6.7)		175 (19.1)	180 (18.1)	
Other	25 (7.6)	16 (4.0)		66 (7.2)	58 (5.8)	
Pre-pregnancy body mass index			0.16			0.005
Underweight (<18.5)	19 (5.9)	14 (3.6)		40 (4.6)	65 (6.8)	
Normal weight (18.5 – 24.9)	160 (49.5)	208 (53.3)		462 (52.6)	543 (56.7)	
Overweight (25–29.9)	86 (26.6)	85 (21.8)		210 (23.9)	217 (22.7)	
Obese (≥30)	58 (18.0)	83 (21.3)		166 (18.9)	133 (13.9)	
Missing*	7	12		39	39	
Maternal education			0.30			0.93
< High school	25 (7.6)	22 (5.8)		145 (15.8)	146 (15.3)	
High school	53 (16.1)	76 (19.6)		219 (23.9)	227 (23.7)	
> High school	252 (76.4)	283 (74.3)		552 (60.3)	583 (61.0)	
Missing		21		1	41	
Number of previous livebirths			0.98			0.13
0	181 (54.9)	215 (54.6)		385 (42.0)	396 (40.0)	
1	97 (29.4)	118 (30.0)		290 (31.6)	356 (36.0)	
≥2	52 (15.8)	61 (15.5)		242 (26.4)	238 (24.0)	
Missing*		8			7	

Table S2 (Cont). Comparison of maternal characteristics between primary analytic sample and individuals excluded for missing DBP measurements, National Birth Defects Prevention Study (2000-2005)

	**Cases**			**Controls**		
	Primary analytic sample	Missing DBP measurements	Fisher's exact test	Primary analytic sample	Missing DBP measurements	Fisher's exact test
**Maternal characteristics**	*n (%)*	*n (%)*	*P-Value*	*n (%)*	*n (%)*	*P-Value*
Family history of hypospadias			0.28			0.44
No	319 (96.7)	381 (94.8)		913 (99.6)	995 (99.8)	
Yes	11 (3.3)	21 (5.2)		4 (0.4)	2 (0.2)	
Study Site			<0.001			<0.001
Arkansas	62 (18.8)	60 (14.9)		157 (17.1)	160 (16.1)	
Georgia	64 (19.4)	85 (21.1)		164 (17.9)	118 (11.8)	
Iowa	28 (8.5)	27 (6.7)		142 (15.5)	112 (11.2)	
Massachusetts	97 (29.4)	128 (31.8)		128 (14.0)	143 (14.3)	
New York	5 (1.5)	41 (10.2)		27 (2.9)	148 (14.8)	
North Carolina	46 (13.9)	16 (4.0)		128 (14.0)	45 (4.5)	
Texas	6 (1.8)	14 (3.5)		127 (13.9)	153 (15.4)	
Utah	22 (6.7)	31 (7.7)		44 (4.8)	118 (11.8)	
Total	330	402		917	997	

*Missing values were not evaluated in Fisher's Exact

Table S3. Association between household DBP concentrations and hypospadias restricted to mothers with complete water-use data, National Birth Defects Prevention Study 2000-2005 (n = 1,213)

DBP	Level	μg/L	Total	Cases	Controls	OR	95% CI	aOR[ϕ]	95% CI
TTHM			1,201						
	Q1	< 37.5		159 (50.0)	441 (49.9)	1.0	REF	1.0	REF
	Q2	≥ 37.5 — < 53.4		93 (29.3)	222 (25.1)	1.2	0.9, 1.6	1.2	0.8, 1.6
	Q3	≥ 53.4		66 (20.8)	220 (24.9)	0.8	0.6, 1.2	0.7	0.5, 1.0
BRF			842						
	Q1	< 0.6		118 (63.1)	327 (49.9)	1.0	REF	1.0	REF
	Q2	≥ 0.6 — < 3.2		52 (27.8)	164 (25.0)	0.9	0.6, 1.3	0.7	0.3, 1.3
	Q3	≥ 3.2		17 (9.1)	164 (25.0)	0.3	0.2, 0.5	0.3	0.1, 0.8
CHLF			842						
	Q1	< 19.9		65 (34.8)	327 (49.9)	1.0	REF	1.0	REF
	Q2	≥ 19.9 — < 35.3		65 (34.8)	164 (25.0)	2.0	1.3, 3.0	1.2	0.8, 2.0
	Q3	≥ 35.3		57 (30.5)	164 (25.0)	1.7	1.2, 2.6	1.1	0.7, 1.7
BDCM			841						
	Q1	< 7.0		77 (41.2)	322 (49.2)	1.0	REF	1.0	REF
	Q2	≥ 7.0 — < 11.0		74 (39.6)	167 (25.5)	1.9	1.3, 2.7	1.9	1.3, 2.9
	Q3	≥ 11.0		36 (19.3)	165 (25.2)	0.9	0.6, 1.4	0.7	0.4, 1.2
DBCM			840						
	Q1	< 2.5		125 (67.2)	327 (50.0)	1.0	REF	1.0	REF
	Q2	≥ 2.5 — < 7.1		42 (22.6)	163 (24.9)	0.7	0.5, 1.0	0.8	0.5, 1.3
	Q3	≥ 7.1		19 (10.2)	164 (25.1)	0.3	0.2, 0.5	0.5	0.3, 1.0
HAA5			928						
	Q1	< 24.5		146 (53.9)	325 (49.5)	1.0	REF	1.0	REF
	Q2	≥ 24.5 — < 37.4		61 (22.5)	166 (25.3)	0.8	0.6, 1.2	0.9	0.6, 1.3
	Q3	≥ 37.4		64 (23.6)	166 (25.3)	0.9	0.6, 1.2	0.8	0.6, 1.2
MBAA			736						
	Q1	0[α]		111 (58.4)	314 (57.5)	1.0	REF	1.0	REF
	Q2	> 0 — < 1.0		16 (8.4)	35 (6.4)	1.3	0.7, 2.4	1.6	0.8, 3.3
	Q3	≥ 1.0		63 (33.2)	197 (36.1)	0.9	0.6, 1.3	0.8	0.5, 1.3
MCAA			736						
	Q1	< 1.3		102 (53.7)	273 (50.0)	1.0	REF	1.0	REF
	Q2	≥ 1.3 — < 3.5		46 (24.2)	136 (24.9)	0.9	0.6, 1.4	0.7	0.4, 1.2
	Q3	≥ 3.5		42 (22.1)	137 (25.1)	0.8	0.5, 1.2	0.7	0.4, 1.2
DBAA			736						
	Q1	< 0.9		111 (58.4)	273 (50.0)	1.0	REF	1.0	REF
	Q2	≥ 0.9 — < 2.1		54 (28.4)	136 (24.9)	1.0	0.7, 1.4	1.0	0.6, 1.6
	Q3	≥ 2.1		245 (13.2)	137 (25.1)	0.4	0.3, 0.7	0.7	0.4, 1.2
DCAA			736						
	Q1	< 12.4		90 (47.4)	273 (50.0)	1.0	REF	1.0	REF
	Q2	≥ 12.4 — < 19.9		50 (26.3)	136 (24.9)	1.1	0.7, 1.7	0.8	0.5, 1.3
	Q3	≥ 19.9		50 (26.3)	137 (25.1)	1.1	0.7, 1.7	0.8	0.5, 1.3
TCAA			736						
	Q1	< 9.4		86 (45.3)	274 (50.2)	1.0	REF	1.0	REF
	Q2	≥ 9.4 — < 15.8		46 (24.2)	135 (24.7)	1.1	0.7, 1.6	0.8	0.5, 1.3
	Q3	≥ 15.8		58 (30.5)	137 (25.1)	1.3	0.9, 2.0	1.0	0.6, 1.5

α Due to low concentration estimates, referent includes all unexposed mothers (i.e., concentration = 0 μg/L)

ϕ Adjusted for: maternal age at conception, study site, parity, maternal education, pre-pregnancy body mass index, and maternal race/ethnicity

Total Trihalomethanes (TTHM); Bromoform (BRF); Chloroform (CHLF); Bromodichloromethane (BDCM); Dibromochloromethane (DBCM); Total Haloacetic Acids (HAA5); Monobromoacetic Acid (MBAA); Monochloroacetic Acid (MCAA); Dibromoacetic Acid (DBAA); Dichloroacetic Acid (DCAA); Trichloroacetic Acid (TCAA)

Table S4. Association between household disinfection by-product (DBP) concentrations above and below US Environmental Protection Agency (EPA) allowable levels and hypospadias restricted to mothers with complete water-use data, National Birth Defects Prevention Study 2000-2005 (n = 1,213)

Regulation categorizations	Cases	Controls	OR	95% CI	aOR$^{\phi}$	95% CI
TTHMs $\leq$ 80 μg/L	295 (92.8)	808 (91.5)	1.0	REF	1.0	REF
TTHMs > 80 μg/L	23 (7.2)	75 (8.5)	0.8	0.5, 1.4	0.6	0.4, 1.1
Total	318	883				
HAA5s $\leq$ 60 μg/L	253 (93.4)	605 (92.1)	1.0	REF	1.0	REF
HAA5s > 60 μg/L	18 (6.6)	52 (7.9)	0.8	0.5, 1.4	0.8	0.4, 1.4
Total	271	657				

ϕ Adjusted for: maternal age at conception, study site, parity, maternal education, pre-pregnancy body mass index, and maternal race/ethnicity

Total Trihalomethanes (TTHMs); Total Haloacetic Acids (HAA5s)
